# Learning to detect boundary information for brain image segmentation

**DOI:** 10.1186/s12859-022-04882-w

**Published:** 2022-08-11

**Authors:** Afifa Khaled, Jian-Jun Han, Taher A. Ghaleb

**Affiliations:** 1 School of Computer Science and Technology, Huazhong University of Science and Technology, Wuhan, China; 2 School of Electrical Engineering and Computer Science, University of Ottawa, Ottawa, Canada

**Keywords:** Medical imaging, Boundary detection, Brain segmentation, *MRI*

## Abstract

MRI brain images are always of low contrast, which makes it difficult to identify to which area the information at the boundary of brain images belongs. This can make the extraction of features at the boundary more challenging, since those features can be misleading as they might mix properties of different brain regions. Hence, to alleviate such a problem, image boundary detection plays a vital role in medical image segmentation, and brain segmentation in particular, as unclear boundaries can worsen brain segmentation results. Yet, given the low quality of brain images, boundary detection in the context of brain image segmentation remains challenging. Despite the research invested to improve boundary detection and brain segmentation, these two problems were addressed independently, i.e., little attention was paid to applying boundary detection to brain segmentation tasks. Therefore, in this paper, we propose a boundary detection-based model for brain image segmentation. To this end, we first design a boundary segmentation network for detecting and segmenting images brain tissues. Then, we design a boundary information module (*BIM*) to distinguish boundaries from the three different brain tissues. After that, we add a boundary attention gate (*BAG*) to the encoder output layers of our transformer to capture more informative local details. We evaluate our proposed model on two datasets of brain tissue images, including infant and adult brains. The extensive evaluation experiments of our model show better performance (a Dice Coefficient (DC) accuracy of up to $5.3\%$ compared to the state-of-the-art models) in detecting and segmenting brain tissue images.

## Introduction

MRI brain images are always of low contrast, which makes it difficult to identify which area the information at the boundary of brain images belongs to. To alleviate such a problem, image boundary detection plays a vital role in medical image segmentation [1, 2], as unclear boundaries can worsen brain segmentation results. Yet, given the low quality of brain images and blurry image boundaries, boundary detection in the context of brain image segmentation remains a research challenge. Results of existing segmentation models can be influenced by blurry image boundaries, which is due to bad boundary pixel differentiation [3]. In brain segmentation, boundary refers to the area that divides brain regions. For example, the dividing area between the white region (*WM*) and grey region (*GM*) of the brain is considered as a boundary. The boundary is crucial in brain segmentation, since if it is unclear, the boundary information between *WM* and *GM* would also be unclear.

**Fig. 1:**
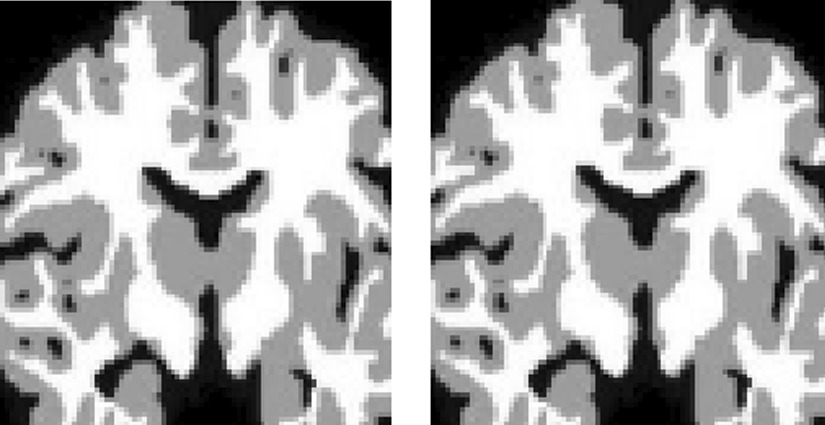
Examples show the ambiguous boundaries between *WM* and *GM*

Despite the research invested to improve boundary detection and brain segmentation, these two problems were addressed independently. Moreover, extracting features at the image boundary remains challenging, since those features can be misleading, since they might mix properties of different brain regions [4]. Many models were proposed to detect or segment human brain tissues [5–7]. Despite the highly reported performance of these models, they suffer from an extreme problem concerning the extraction of local details in ambiguous boundaries [8–10]. Much research has addressed such a problem [8, 11, 12]. Traditional methods that are atlas-based are not accurate and not robust [13]. Also, deep learning models were introduced to address this problem, yet, ambiguous boundaries have not been sufficiently resolved. What complicates the detection of image boundaries for brain tissues segmentation is the low contrast and unclear boundaries between *WM* and *GM*. Figure 1 shows an example of ambiguous boundaries between *WM* and *GM*.

Therefore, in this paper, we propose a boundary detection-based model for brain image segmentation. In particular, we focus on the boundary information between *WM* and *GM*, especially for low contrast images. First, we design a boundary segmentation network for detecting and segmenting brain tissues. Second, we design a boundary information module (*BIM*) to help distinguish between the boundaries of three different brain tissues. Finally, we add a boundary attention gate (*BAG*) to each output layer of the encoder of our transformer to capture more informative local details. We evaluate our proposed model on two datasets of brain tissue images: infant and adult brains. Our model achieves higher results (i.e., a Dice Coefficient (DC) accuracy of up to $5.3\%$) compared to the state-of-the-art models. In addition, our model is less complex and performs faster than the state-of-the-art models. In summary, this paper makes the following contributions:

- We design a network model that performs both boundary detection and brain tissues segmentation to improve the segmentation accuracy.
- We design a boundary information module (BIM) to distinguish the boundaries of different brain tissues.
- We design a boundary attention gate (BAG) to capture more local details about brain tissues.

The rest of this paper is organized as follows. Section 2 presents the prior models related to the boundary detection of brain segmentation. Section 3 presents the design of our proposed model. Section 4 presents our experimental design and evaluation. Section 5 presents our evaluation results and discusses the strengths and limitations of our model. Finally, Sect. 6 concludes the paper and discusses future work.

## Related work

This section reviews the state-of-the-art techniques for boundary detection and brain segmentation. In Table 1, we provide a summary of the recent works in medical imaging.

### Boundary detection

Boundary detection has recently been an active research problem for which many techniques have been proposed to extract boundary information, thus mitigating the problem of ambiguous boundaries [14–16]. However, the problem of unclear boundaries between (*WM*) and (*GM*) remains challenging due to the low contrast of MRI images. This problem has also been studied extensively [17–19]. The main focus of these studies was on mixed features between *WM* and *GM*, in which the boundary information of these two regions is unclear and hard to identify. Specifically, the research conducted in [12, 20–22] focused on skin lesions segmentation from dermoscopy images in which the contrast between the lesion and normal skin is fairly low. Features used in [12, 21, 22] to detect boundaries achieved a significant improvement to the state-of-the-art techniques. To deal with the global context to segment lesion from normal skin, Blackmon et al. [8] proposed a model to help segmenting lesions. To improve boundary detection results, whereas Andrews et al. [9] proposed a novel unsupervised pre-training framework using boundary-aware preserving learning.

Despite the effort invested in boundary detection, little attention was paid to applying it to brain tissues segmentation, which is usually affected by unclear boundary areas.

**Table 1:** Summary of the state-of-the-art techniques in medical image

Publication	Method	Purpose
Guoqiang et al. [23]	*GVF*	Segmentation of brain MRI image with GVF snake model
Lei et al. [24]	Clustering method	MR brain image segmentation
Somasundaram et al. [25]	Intensity thresholding	Brain portion segmentation from MRI
Jiao et al. [26]	$MI-GAN$	Brain image segmentation based on bilateral symmetry information
Jimenez et al. [27]	3*DCycleGAN*	Data-driven brain MRI segmentation supported on edge confidence and a priori tissue information
Tan Ou et al. [28]	Atlas	Automatic segmentation of human brain images
Snell et al. [29]	Active surfaces	Model-based segmentation of the brain from 3-D MR
Lei et al. [24]	Clustering method	MR brain image segmentation
Yao et al. [30]	Adjustable method	High effective medical image segmentation
Zhang et al. [31]	Active volume model with shape priors	3D segmentation of rodent brain structures
Liya et al. [32]	Object detection	Feature extraction and morphological operations
Mallick et al. [33]	Intelligent technique	*CT* brain image segmentation
Zhou et al. [34]	Encoder–decoder networks	Low-contrast medical image segmentation
Qu et al. [35]	FCD detection	Estimating blur at the brain gray-white matter boundary
Shen et al. [36]	Fully convolutional networks	Neuronal boundary detection
Chakraborty et al. [37]	An integrated approach	Boundary finding in medical images
Khaled et al. [17]	3D, FCN + MIL + G + K	Brain tissues segmentation
Khaled et al. [38]	Multi-stage GAN	Brain tissues segmentation

### Brain segmentation

There have been many proposed models (e.g., [38, 39]) for brain tissues segmentation. These models divided the brain image into multiple regions. For example, [40, 41] divided the brain into eight regions), whereas [42, 43] divided the brain into three regions. Dolz et al. [44] proposed 3*D* and fully *CNN* for the segmentation of the subcortical brain structure. Later on, Bao and Chung [7] have improved the model proposed by Dolz et al. using a multi-scale structured *CNN* with label consistency. Jin et al. [45] have also proposed *CNNs* models with the use of residual connections to segment white matter hyperintensity from *T*1 and flair images. Their models outperformed previous models with an overall dice coefficient of 0.75% on *H*95 and 27.26% on an average surface distance. Fechter et al. [6] also used fully *CNNs* for brain segmentation. Using five datasets, they obtained dice coefficient ranging between 0.82 and 0.91 for each dataset. de Brebisson and Montana [46] proposed a random walker approach driven by a 3*D* fully *CNN* for different tissue classes. Their model was able to segment the esophagus using *CT* images. Ma et al. [47] proposed a visual detection of cells in brain tissue slice for patch clamp system.

Khaled et al. proposed two brain tissues segmentation models, one using FCN + MIL + G + K [17] and another using a multi-stage GAN model [38]. They evaluated their models on two infants and adults brain images and obtained good segmentation results, expressed by dice coefficients of up to 94% for segmenting GM and WM.

Despite the effort invested in brain tissue segmentation, segmentation results still suffer from mixed tissue information caused by unclear image boundaries, which confuses models in precisely identifying what features belong to which region of the brain.

### Highlights on related work

Unlike previous work, our objective in this paper is to solve the problem of unclear boundaries in brain segmentation. In particular, the state-of-the-art techniques either performed boundary detection or image segmentation, independently, thus not considering the fusion of both detection and segmentation in one model. Hence, in this paper, we design a boundary segmentation network for detecting and segmenting images of brain tissues. Then, we design a boundary information module (*BIM*) to distinguish boundaries from the three different brain tissues. After that, we add a boundary attention gate (*BAG*) to the encoder output layers of our transformer to capture more informative local details.

## Method

**Fig. 2:**
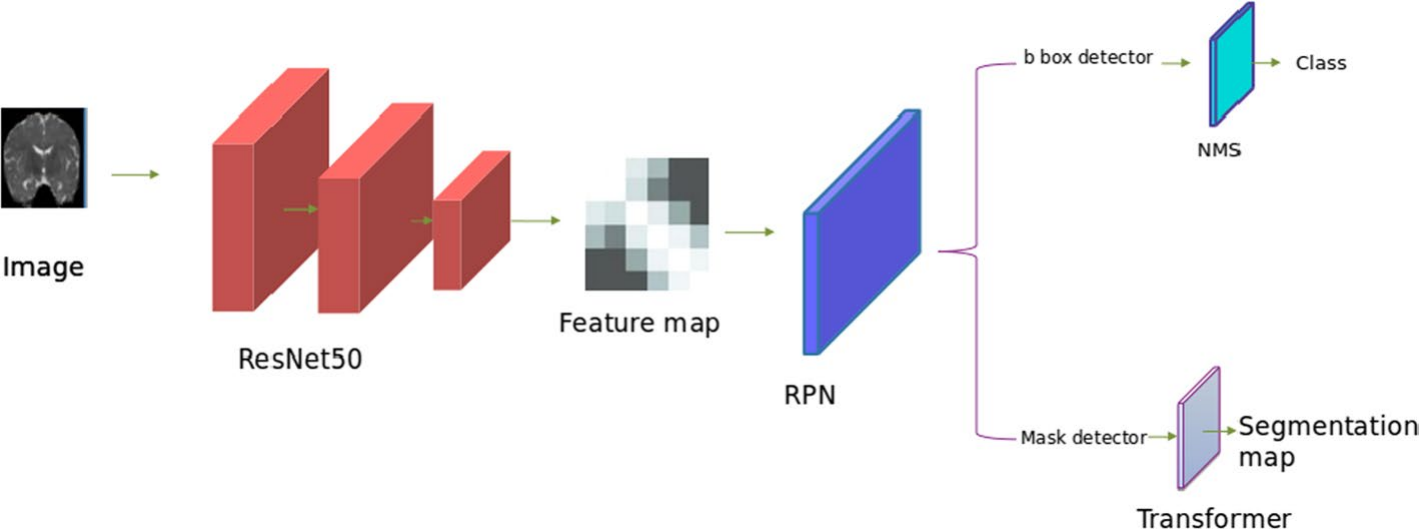
An overview of the proposed model

**Fig. 3:**
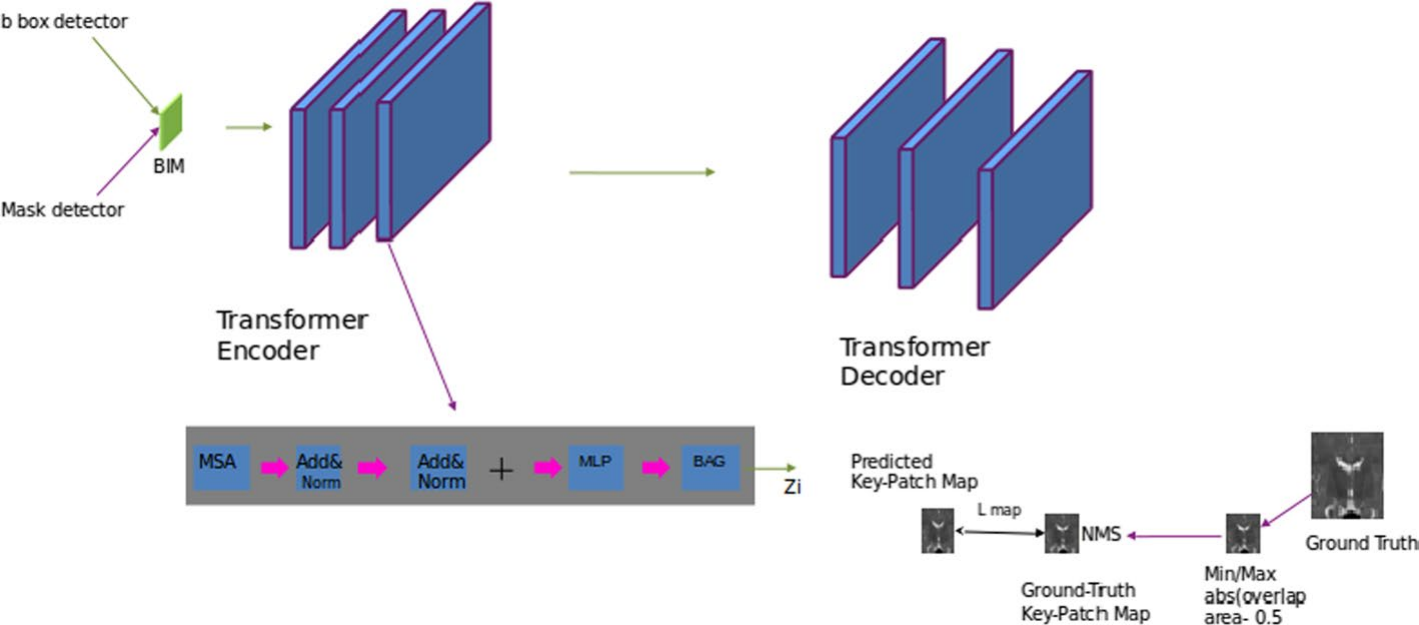
The architecture of our model’s transformer

We propose a model in which we take advantage of the connection between both boundary detection and brain segmentation. To this end, we design a boundary segmentation network for the detection and segmentation of brain tissues. Then, we design the boundary information module (*BIM*) to distinguish boundaries of the three different brain tissues. Figure 2 gives an overview of architecture of our proposed model. We use the *ResNet*50 network [48] to extract feature maps from input images. Inspired by the excellent success of region proposal networks (*RPN*), we use it in our model to generate a bbox detector and mask detector. Then, the model has two branches: one for detection, which follows the non maximum suppression (*NMS*), and another for segmentation, which follows the transformer whose architecture is shown in detail in Fig. 3. Table 2 lists all the symbols we refer to in this paper.

**Table 2:** List of symbols referred to in this paper

Symbol	Definition
*WM*	White matter
*GM*	Gray matter
*CSF*	Cerebrospinal fluid
*Conv*	Convolutional
*LeReLU*	Activation function
*E*	Expected value
*DC*	Dice Coefficient
*MRI*	Magnetic resonance imaging
*T*1	Subject-1-to-subject-10
*T*	Subject-11-to-subject-23
$V_auto$	Automated segmentation
$V_ref$	Reference segmentation
*BIM*	Boundary information module
*DICE*	Dice loss function
*CE*	Cross-entropy loss function

### Boundary information module (BIM)

Feature maps are obtained from the segmentation branch and detection branch, and *R* channels are consider. Feature maps are divided into groups *M* where each group maintains a vector at every position.

1 $$\begin{aligned} X = \{{x_{\text {1}}^{cls}, \ldots , x_{\text {s}}^{cls}}\}, \; x_{\text {i}}^{cls} \in R^{C/G} \end{aligned}$$

The global statistical feature is used to approximate the vector by a spatial averaging function, $(F_{\text {gp}})$, as follows.

2 $$\begin{aligned} g = F_{\text {gp}} =1/s \sum \limits _{{\text {i=1}}}^{s}x_{\text {i}}^{mask}, \end{aligned}$$

To measure the similarity between vectors and features, we generate the correlation coefficient, $(c_{\text {i}})$, as follows.

3 $$\begin{aligned} c_{\text {i}} =||g|| \; ||x_{\text {i}}^{cls}|| \; \cos (\theta {\text {i}}) \end{aligned}$$

Normalization is then used to avoid the biased magnitude of $c_{\text {i}}$, as follows.

4 $$\begin{aligned} \bar{c_{\text {i}}}= c_{\text {i}} - \mu _{\text {c}}/\sigma _{\text {c}}+\epsilon , \end{aligned}$$

where $$\epsilon =1e-6$$.

Two parameters, $\alpha$ and $\beta$, are used to represent the identification and localization of features, as follows.

5 $$\begin{aligned} a_{\text {i}}= & {} \alpha \bar{c_{\text {i}}} + \beta , \end{aligned}$$

6 $$\begin{aligned} X_{\text {i}}^{mask}= & {} x_{\text {i}}^{mask} \; . \; \sigma (a_{\text {i}}), \end{aligned}$$

where $x_{\text {i}}^{mask}$ denotes the segmentation feature vector and $\sigma$ denotes the sigmoid function.

The output of *BIM* is represented as follows.

7 $$\begin{aligned} X= \{x_{\text {i}}^{mask}, \ldots , x_{\text {s}}^{mask}\}, \; x_{\text {i}}^{mask} \in R^{c} \end{aligned}$$

### Loss functions

Loss functions are related to two parts: the boundary detection part and the segmentation part. A *Dice* loss function $(\Phi DICE)$ is used to reduce the difference between the ground truth and the segmentation map $(L_{seg})$. A cross-entropy loss function $(\Phi CE)$ is used to minimize the difference between the ground truth and predicted-key map $(L_{Map})$.

8 $$\begin{aligned} l_{\text {seg}}= & {} \Phi DICE(S_{\text {GT}},S_{\text {pred}}), \end{aligned}$$

9 $$\begin{aligned} l_{\text {Map}}^{i}= & {} \Phi DICE(M_{\text {GT}},M_{\text {pred}}), \end{aligned}$$

where $S_{\text {GT}}$ is the ground truth and $S_{\text {pred}}$ is the segmentation map.

10 $$\begin{aligned} L_{\text {whole}} = \sum \limits _{{\text {i=1}}}^{n+1}l_{\text {Map}}^{i} +L_{\text {seg}}, \end{aligned}$$

where $M_{\text {GT}}$ is the ground truth key patch map and $M_{\text {pred}}$ is the predicted-key map.

### Boundary aware transformer

To improve boundary detection and the extraction of boundary information in brain segmentation with ambiguous boundaries, we use a transformer, in which a *BAG* is added to the end of its encoder layer. As shown in Fig. 2, *BAG* consists of a key patch map generator. The generator takes the transformed feature as input and generates a binary patch map as output. The boundary-aware transformed feature is represented as follows.

11 $$\begin{aligned} {V}^{i-1}= MSA (Z^{i-1}) + MLP (MSA(Z^{i-1})), \end{aligned}$$

12 $$\begin{aligned} {Z}^{i}= V^{i-1} + (V^{i-1}* \hat{M}^{i-1}), \end{aligned}$$

where $+$ and $*$ denote the element-wise addition and channel-wise multiplication, respectively.

## Experiments

This section presents our experimental design and evaluation. First, we give a more detailed description of the datasets used in our experiments. Then, we describe the Dice Coefficient (*DC*) of the segmentation evaluation. Finally, we describe our experimental setup.

### Overview of the datasets

#### Datasets

In our experiments, we use two datasets for evaluating our model: the $MICCAI\ iSEG$ infant dataset and *MRBrainS* adult dataset. The MICCAI iSEG-2017 dataset contains training and testing data of 6-month infants, whereas the MRBrainS-2013 dataset contains training and testing data for adults. The two datasets are obtained from different organizations, and there are significant differences between images in the infant dataset and the adult dataset in terms of image data characteristics, such as the bunch of tables images and the number of available modalities. In addition, both datasets were used to evaluate the previous models in this context.

#### The MICCAI iSEG-2017 dataset

**Fig. 4:**
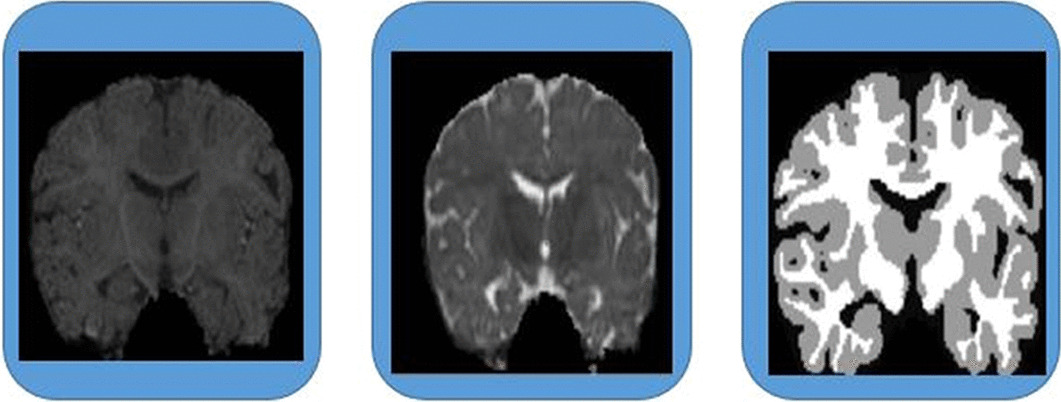
An example of the $MICCAI\ iSEG$ dataset (*T*1, *T*2, manual reference contour)

The aim of the evaluation framework^1^ introduced by the MICCAI iSEG organizers is to compare segmentation of *WM*, *GM* and *CSF* on *T*1 and *T*2. The training dataset contains 10 images, named *T*1-1 through *T*1-10, *T*2-2 through *T*2-10, and a ground truth. The testing dataset contains 13 images, named *T*-11 through *T*-23. Figure 4 shows an example of the $MICCAI\ iSEG$ dataset. Table 3 shows the parameters used to create *T*1 and *T*2. Two different times were used to create *T*1 and *T*2, which are the longitudinal relaxation time and transverse relaxation time.

#### The MRBrainS-2013 dataset

The *MRBrainS* dataset^2^ contains 20 subjects on *T*1, *T*2, and *FLAIR*. The dataset contains five subjects for as a training set and 15 subjects as a testing set. In this dataset, adult brain images has multiple regions to segment, including (a) white matter lesions, (b) basal ganglia, (c) lateral ventricles, (d) cortical gray matter, (e) peripheral cerebrospinal fluid, (f) white matter, (g) cerebellum, and (h) brain stem.

**Table 3:** Parameters used to generate *T*1 and *T*2

Parameter	*TR*/*TE*	Flip angle	Resolution
*T*1	1900/4.38 ms	7	1$\times$1$\times$1
*T*2	7380/119 ms	150	1.25$\times$1.25$\times$1.25

#### Dice coefficient (DC)

We use the Dice Coefficient (*DC*) metric for evaluating our model. This metric assesses how effective and robust the model is. *DC* has been widely used as a benchmark in the literature to compare brain segmentation models. The *DC* is given by the following equation (defined in [49]):

13 $$\begin{aligned} {DC}(V_{\text {ref}}, V_{\text {auto}}) = \frac{2 V_{\text {ref}} \bigcap V_{\text {auto}} |}{|V_{\text {ref}}| + |V_{\text {auto}}|} \end{aligned}$$

where $V_{\text {ref}}$ denotes for the reference segmentation, $V_{\text {auto}}$ denotes for the automated segmentation. *DC* values are given in the range of [0, 1], where 1 denotes a perfect overlap and 0 denotes a complete mismatch.

#### Experiment environment

We implement our proposed model using Python TensorFlow on a computer with a *NVIDIA* GPU and the Ubuntu 16.04 operating system. We train and test our model on each of the two datasets independently.

## Results and discussion

This section discusses the evaluation results of our model compared to the state-of-the-art models.

### Analysis of the results

Table 4 shows the performance of our model on the *MICCAI* *SEG* dataset, compared to the state-of-the-art models. The results show that our model achieved high results compared to the state-of-the-art models. In particular, we observe an increase in the accuracy of segmenting the *GM* using our model. This result suggests that *BIM* has contributed the improved distinction between the boundaries for *GM*. However, for segmenting *CSF* and *WM*, we observe that the result of our model was $1\%$ lower than those proposed in [17] and [38], which is likely due to the inclusion of some irrelevant information of the *GM* in *CSF* and *WM*. This encourages us to further improve the boundary detection to carefully account for the features missed by our current model. Besides, we plan in the future to apply boundary detection to multi-stage segmentation models, given their current high accuracy even when no boundary detection is adopted.

**Table 4:** Segmentation performance in Dice Coefficient (*DC*) obtained on the $MICCAI \ iSEG$ dataset achieved by our model (with and without *BIM*), compared to the state-of-the-art models

Model	Dice Coefficient (DC) accuracy		
	CSF (%)	GM (%)	WM (%)
Özgün et al. [50]	91.2	86.1	84.1
Dong et al. [51]	83.5	85.2	86.4
Konstantinos et al. [51]	90.3	86.8	84.3
Mahbod et al. [52]	85.5	87.3	88.7
3D, FCN + MIL + G + K [17]	94.1	90.2	89.7
Multi-stage [38]	**95.0**	94.0	**92.0**
Ours (with *BIM*)	94.0	**94.3**	91.0
Ours (without *BIM*)	90.0	89.0	86.0

The best performance for each tissue class is highlighted in bold

**Fig. 5:**
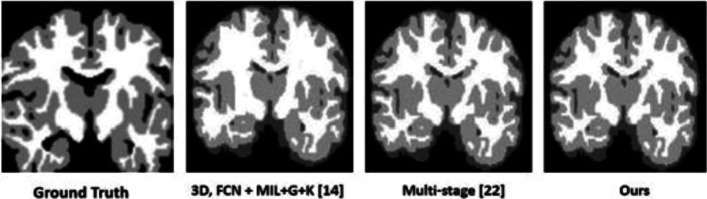
Visualization results on *MRBrainS* dataset

Table 5 shows the performance of our model on the MRBrainS dataset, compared to the state-of-the-art models. We observe an increase in the accuracy of segmenting both the *GM* and *WM* using our model. This result suggests that *BIM* has contributed the improved distinction between the boundaries for the *GM* and *WM*. Once again, we observe that our model performs $1\%$ lower than the multi-stage model in segmenting *CSF*, thus suggesting a limitation of our boundary detection at that region of the brain. Figure 5 visualizes the results of our model on the images used as a validation set. As we can see, the segmentation results achieved by our model are fairly close to the manual reference contour (i.e., ground truth) provided by the MICCAI iSEG organizers. Additionally, we observe an improvement of segmentation accuracy between *WM* and *GM*.

**Table 5:** Segmentation performance in Dice Coefficient (*DC*) obtained on the *MRBrainS* dataset achieved by our model (with and without *BIM*), compared to the state-of-the-art models

Model	Dice Coefficient (DC) accuracy		
	CSF (%)	GM (%)	WM (%)
Özgün et al. [50]	83.9	88.9	89.4
Dong et al. [51]	83.5	85.4	88.9
Mahbod et al. [52]	85.5	87.3	88.7
Marijn et al. [53]	85.5	87.3	88.7
3D,FCN+MIL+G+K [17]	**94.1**	90.2	89.7
Multi-stage [38]	93.0	93.0	88.0
Our model (with *BIM*)	92.0	**95.0**	**93.0**
Our model (without *BIM*)	89.0	90.0	90.0

The best performance for each tissue class is highlighted in bold

### Ablation experiment

In the context of research, where deep learning is employed, an *ablation experiment* is important to describe a model and give a better understanding of the model’s performance. The ablation study helps reveal the effectiveness of *BIM* in our model.

*Effectiveness of BIM* To demonstrate the effectiveness of *BIM*, we run our model without *BIM* on both datasets and compare the results with the state-of-the-art models in the last rows of Tables 4 and 5. We observe that *BIM* helped our model distinguish between the boundaries of the three brain tissues. In particular, *BIM* improved segmentation accuracy by 4.0–5.3%.

### Execution time

Table 6 shows the execution time (in minutes) and the standard deviation (*SD*) for our model on the *MRBrainS* dataset, compared to the state-of-the-art models. We observe that our model is faster than all the state-of-the-art models, except one where our model took a few minutes long. We conjecture that such longer execution time is likely due to the additional steps required for boundary detection, which added some level of complexity to proposed model. Still, given the better segmentation results of our model, accuracy should be given more preference than efficiency, since the gap in execution time is not considerably large.

**Table 6:** Average execution time (in minutes) and standard deviation (*SD*) on the *MRBrainS* dataset

Model	Time (*SD*)
Özgün et al. [50]	15.40 (0.16)
Dong et al. [51]	19.23 (0.20)
Mahbod et al. [52]	17.6 (0.18)
Marijn et al. [53]	18.4 (0.15)
3D, FCN + MIL + G + K [17]	**5.9 (0.11)**
Multi-stage [38]	22.61 (0.21)
Our model (with *BIM*)	10 (0.3)
Our model (without *BIM*)	9 (0.14)

The fastest model is highlighted in bold

### Highlights of our model

*Boundary detection for brain segmentation* To the best of our knowledge, our proposed model is the first attempt to apply boundary detection for the segmentation of brain tissues, which has shown a significant improvement to segmentation results. Our model outperformed previous models not only in terms of segmentation accuracy, especially for segmenting GM and WM, but also in terms of execution time.

*BIM+BAG* Our model adopts the *BIM* and *BAG* mechanisms to focus on boundaries while performing the segmentation tasks. The $BIM+BAG$ addition to our model shows a positive effect to the effectiveness of our model. Still, these two mechanisms may have introduced some level of complexity to our model, but still performs faster than all the state-of-the-art models, except one. Nevertheless, we believe that more preference should be given to producing better segmentation results regardless of execution time. Hence, sacrificing efficiency for a better accuracy is a viable option.

*Accuracy on two different datasets* Our model is evaluated on two completely different datasets of brain images, one for infants and one for adults. Each of these datasets contains a limited number of images with low contrast. Yet, our models shows high results for segmenting brain tissues, most particularly the *GM* and *WM*, which outperformed the state-of-the-art models in this context.

### Limitations and future work

*Limited dataset* Our model is evaluated on datasets including infant and adult images. However, these images are limited and of poor quality, which could have influenced the performance of our model. Future research should consider extending the evaluation of boundary detection+segmentation on additional, more realistic datasets.

*Network design* Our model employs ResNet50 to extract feature maps from input images and RPN to generate a bbox detector and mask detector. However, these networks might not be the best alternative for this particular problem. Future work should investigate other networks (CNN, RNN, Unit Network, etc.)

*Further improvement of boundary detection* Our models achieved a higher performance, compared to the state-of-the-art models, for segmenting GM and WM. However, the performance of our model compared to the multi-stage model was lower on CSF. This indicates that there is still room for improve segmentation accuracy by considering more sophisticated boundary detection and/or applying it to other segmentation models.

*Model complexity* It can be argued that our model has become more complex with the additional networks and layers employed to perform boundary detection followed by tissue segmentation. However, our model shows better efficiency, expressed by the faster execution times compared to the state-of-the-art models. Still, we aim in the future to optimize our model further to mitigate the accuracy versus efficiency trade-off by reducing any level of complexity.

## Conclusion

In this paper, we proposed a boundary detection-based model for brain image segmentation. To this end, we designed a boundary segmentation network for detecting and segmenting brain tissues. Then, we designed a boundary information module (*BIM*) to distinguish boundaries from the three different brain tissues. After that, we added a boundary attention gate (*BAG*) to the encoder output layers to capture more informative local details. We evaluated our proposed model on two datasets of brain tissue images, including infant and adult brains. Our evaluation results of our model show better performance (a Dice Coefficient (DC) accuracy of up to $5.3\%$ compared to the state-of-the-art models) in detecting and segmenting brain tissue images, which proves the importance of boundary detection for brain segmentation tasks.

We plan in the future to expand the evaluation of our model to consider additional datasets with more brain images and tissues. We also plan to extend our model to perform segmentation of pathological brain and skin lesion dermoscopy images. Moreover, we plan to investigate other networks than *RPN* (e.g., Cascade Mask $R-CNN$ networks) to further improve segmentation accuracy. Finally, We plan to develop a framework to support boundary detection in other segmentation models.

## Data Availability

The data that supports the findings of this study is available at MICCAI Grand challenge on 6-month infant brain *MRI* segmentation (http://iseg2017.web.unc.edu) and MRBrains (https://mrbrains13.isi.uu.nl/results.php) and are both publicly available.

## Abbreviations

G: Generator
D: Discriminator
z: Noise
G(z): Generated data
x: Real data
WM: White matter
GM: Gray matter
CSF: Cerebrospinal fluid
Conv: Convolutional
LeReLU: Activation function
GAN: Generative adversarial network
E: Expected value
DC: Dice Coefficient
MRI: Magnetic resonance imaging
T1: subject-1-to-subject-10
T2: subject-11-to-subject-23
Vauto: Automated segmentation
Vref: Reference segmentation

## Footnotes

1 http://iseg2017.web.unc.edu.

2 https://mrbrains13.isi.uu.nl/results.php.
